# Reducing Opioid Overprescribing by Educating, Monitoring and Collaborating with Clinicians: A Quality Improvement Study

**DOI:** 10.7759/cureus.7778

**Published:** 2020-04-22

**Authors:** Avni Gupta, Stephanie Lindstrom, Gauri Shevatekar

**Affiliations:** 1 Pain Management, Unitypoint Health Methodist, Peoria, USA; 2 Family Medicine, Unitypoint Clinic Methodist, Peoria, USA; 3 Graduate Medical Education and Family and Community Medicine, University of Illinois College of Medicine, Peoria, USA

**Keywords:** cdc opioid guidelines, monitoring opioid prescribing patterns, provider education and collaboration, opioid taper, patient satisfaction

## Abstract

Introduction

Multiple barriers have been described for reducing opioid prescribing by primary care providers. We describe a quality improvement report on the effects of a series of focused interventions on opioid prescribing after the release of the Centers for Disease Control and Prevention (CDC) guidelines while monitoring patient satisfaction.

Material and methods

The study began as an intervention project to inform and educate providers about the CDC’s guidelines and to improve adherence. A convenience sample of 165 providers from 33 outpatient clinics of a healthcare system was utilized. This quality improvement study compared a 20-month preintervention baseline period with a 16-month post-intervention period ending on December 31, 2017, using the health system’s electronic medical record. Interrupted time series analysis was used to assess the effect of the intervention on opioid prescribing. Providers were given quarterly individual reports on their prescribing patterns of schedule II opioids and comparing their prescribing patterns to their peers. Providers had access to educational opportunities for CDC guidelines, various aspects of safe opioid prescribing, and professionally written patient hand-outs about opioid risks and alternatives. Provider collaboration with patients for tapering opioids and collaboration with specialists in managing complex pain patients was encouraged. A total number of schedule II chronic opioid prescriptions per month was measured.

Results

The total schedule II opioid prescription rate was 19.6% lower than the average of the baseline. Every month after August 2016, there was a significant reduction of total schedule II opiate orders with a risk decrease of 2% [risk ratio (RR) 0.982; 95% confidence interval (CI) 0.976-0.989; p < 0.0001]. The patient satisfaction scores improved from 92.1 % in January 2015 to 95.1% by December 2017.

Discussion

We noticed an initial decrease in opioid prescribing with the release of the CDC guidelines. However, a greater decline in opioid prescribing was noted after distributing data to providers that compared their own opioid prescribing patterns to their peers. This data offered an opportunity for self-analysis to clinicians to justify the clinical reasons for writing more opioid prescriptions. Provider and patient education on the benefits of opioid reduction enabled better collaboration and engagement in shared decision making with a detailed plan of gradual opioid reduction. Our study was limited by the inability to determine the most effective intervention as the interventions were initiated as a bundle in our healthcare system. Indications for opioid therapy such as pain management for cancer pain or palliative care versus chronic non-cancer pain were not available. The major adverse events related to opioid use, such as opioid overdose deaths and opioid use disorder, were not measured in this data source.

Conclusions

Opioid overprescribing was reduced by educating providers and patients, monitoring clinicians’ opioid prescribing patterns, and seeking physicians’ collaboration. Future healthcare initiatives can utilize similar methods to evaluate interventions impacting the opioid epidemic.

## Introduction

Chronic opioid therapy for non-cancer pain has increased exponentially in the last two decades without evidence of significant improvement in pain control and function [1]. This is in conjunction with an associated increase in disability, healthcare costs, malpractice claims, and opioid overdose-related deaths [2-3]. Communities with higher rates of opioid prescriptions suffer from higher opioid-related adverse events including deaths, even in individuals without prescriptions, possibly due to diversion [4-8]. Hence, interventions to reduce the opioid prescribing need to be explored to influence the opioid epidemic.

The majority of controlled substance prescriptions are generated in the outpatient settings. Providers in primary care and internal medicine along with dentists are known to be the highest prescribers of controlled substances [9-12]. In addition, nurse practitioners and physician assistants have been shown to prescribe opioids at higher rates than the physicians [13]. Lack of adequate training on chronic pain management and/or opioid addiction can be seen as one of the factors behind higher opioid prescribing rates seen among them [14-15].

In March 2016, the Centers for Disease Control and Prevention (CDC) released the guidelines for providers who prescribe opioids for non-cancer pain [16]. The guidelines focused on primary care providers to address the pertinent issues including when to initiate, continue and end opioids in the treatment of chronic non- cancer pain, as well as the dosage, selection, duration and assessment of the risks and harms of opioid use. However, the ability of guidelines to change practice behavior might be limited [17-18]. After the release of the CDC guidelines, it is observed that primary care clinicians face multiple barriers in reducing opioid prescriptions including difficulty in tapering opioids in patients who are on long term chronic opioids and lack of access to adjunctive management strategies [19].

Here, we describe a quality improvement report which focuses on collaboration, education to providers, and monitoring of the providers’ opioid prescribing patterns as an intervention as well as an assessment of patient satisfaction. This study utilized previously collected data by the locally formed Opioid Task Force. To our knowledge, we are not aware of any data published using a similar process to impact the opioid epidemic by influencing prescribing patterns. After the publication of CDC guidelines on opioid prescriptions, a multidisciplinary Opioid Task Force was formed in early 2016 within the Unity Point Health Healthcare System based in Peoria, IL. The Opioid Task Force consisted of seven physicians and two administrators. These included the Executive Medical Director of Primary Care, the Executive Medical Director of Specialty, the Vice President of Medical Affairs, a Rheumatologist, the Medical Director for the Pain Clinic, the Medical Director for the Addiction and Recovery Center and an Attending Physician for the Family Medicine Residency. The two additional administrators were the Vice President of Operations in Specialty and Primary Care. The task force met once every month or once every two months over the course of two years to gauge progress. Goals of the task force were:

a) To educate the providers on the CDC guidelines, safe opioid prescribing and utilization of urine toxicology

b) To assess the current patterns of providers’ opioid prescribing behaviors

c) To monitor and evaluate changes (reduction) in the opioid prescribing behavior of the providers.

d) To assess patient satisfaction.

## Materials and methods

This quality report conforms to the Standards for Quality Improvement Reporting Excellence (SQUIRE) reporting guideline for healthcare safety innovations. The study began as an intervention project to inform and educate providers about the CDC’s guidelines and to improve adherence. A convenience sample of 165 providers from 33 outpatient clinics, serving in the UnityPoint Health in Peoria, IL, was utilized. The majority of these providers were practicing primary care with the exception of two rheumatologists, three neurologists, and two podiatrists. Providers excluded were pain management specialists as they were not employed by the hospital. New providers were added to the data as they entered the medical group. Data consisted of prescriptions written for the most commonly prescribed schedule II opioids including fentanyl, morphine, hydrocodone, hydromorphone, methadone, and oxycodone, over three consecutive months (chronic opioid prescriptions). A unique patient was defined as a single patient who was allocated to a provider in the medical group for two years or more. All clinical activity data was derived from Epic, which is the health system’s electronic medical record.

Each provider was assigned a random identification number which corresponded to his or her prescribing data of chronic opioid prescriptions. Chronic opioid prescriptions were graphed by month. This data was given to each provider in an envelope with their personal identifier to view their prescribing patterns on a graph which showed all providers identified by their random numbers (see the Appendix for Figure 5). This allowed the provider to view and compare his/her own data against their colleagues without any identifying information. Baseline data were obtained from January 2015 to August 2016. At the time of baseline data distribution, all providers were presented the newly released CDC guidelines for opioid prescribing. A patient education handout (created and approved by members of our Opioid Task Force) explaining the guidelines and benefits of the opioid reduction was distributed to the providers’ offices (see the Appendix for Figure 6). The handout also explained collaboration with the provider for a gradual taper and development of a comprehensive pain management plan. This handout carried the corporation logo to show cohesiveness amongst the clinics. Each provider was updated quarterly with their individual data starting from August 2016 till December 2017. The data of the overall decline in opioid prescribing in the healthcare system was shared with the medical group quarterly to report the progress and to further motivate the providers in the ongoing effort to improve opioid prescribing patterns.

The providers were informed further during an “All Provider” meeting. The presentations were focused on: a) giving the providers the resources and education to manage their opioid prescribing and b) providing them the administrative support during this difficult time of transition in prescribing practices. The lecture topics included urine toxicology, newly released CDC guidelines, alternative options for management of chronic pain, and an opioid tapering and withdrawal. Presenters were a pathologist, a pain specialist, and an addiction specialist. Resources including the Pain Clinic, Addiction Recovery Center and local suboxone clinics were formally introduced and discussed by the Executive Medical Director of Primary Care. Incorporation of the Opioid Risk Tool into the EMR was recommended to help identify high-risk individuals and encourage multispecialty collaboration. A “Safe Opioid Prescribing” handout was distributed to providers emphasizing the importance of identifying and treating the cause of the pain as well as recommended documentation in compliance with the CDC guidelines. A controlled substance agreement completed at baseline and updated annually with documentation in a standardized location on the patient problem list for visibility to other care team members was listed as a requirement. CME opportunities for safe opioid prescribing were provided to clinicians during this reporting period to encourage maximum participation in the educational events.

To account for any changes in clinical volumes that might affect opioid volume, we collected the data on number of unique patients using standard health care system reports. Patient Satisfaction was monitored during this timeframe via Press Ganey Surveys. This data was available to providers on a monthly basis via dashboards that were distributed to the providers at each clinic by the clinic administrators. The data showed the individual providers’ patient satisfaction based on physician communication quality and the likelihood of recommending the provider.

This study was designated as “not human subject research” by the University Of Illinois College Of Medicine at Peoria (UICOMP) IRB as it contained coded data with no identifiable information.

Statistical Analysis

This report consists of interrupted time series analyses to examine changes in opioid prescribing. This method has been found to be useful in the evaluation of population-level interventions in which randomization is not possible and no control group is available [20]. This method may be used to determine whether the time at which a new policy or program was implemented is associated with changes in outcome and is measured continuously over time. It may have stronger validity due to a more detailed assessment of the longitudinal impact of an intervention in real-world settings [21]. Analysis was done using R version 3.1.1.

## Results

**Figure 1:**
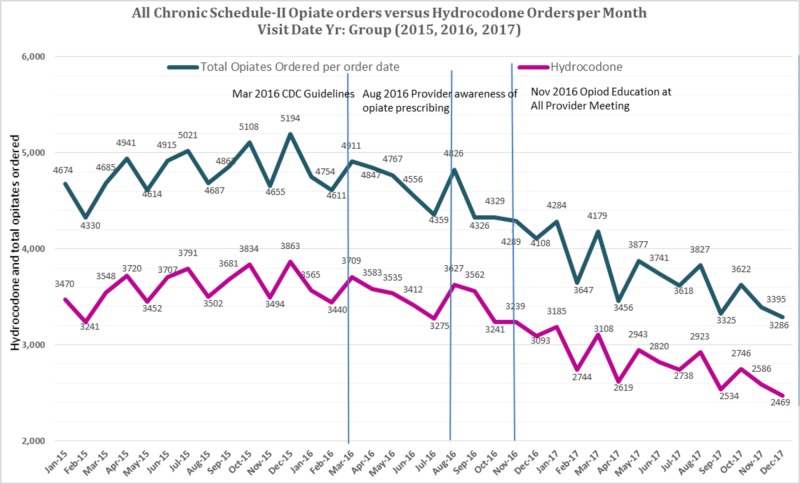
Total Schedule II Opiate orders and Hydrocodone Orders per month from 2015-2017

**Figure 2:**
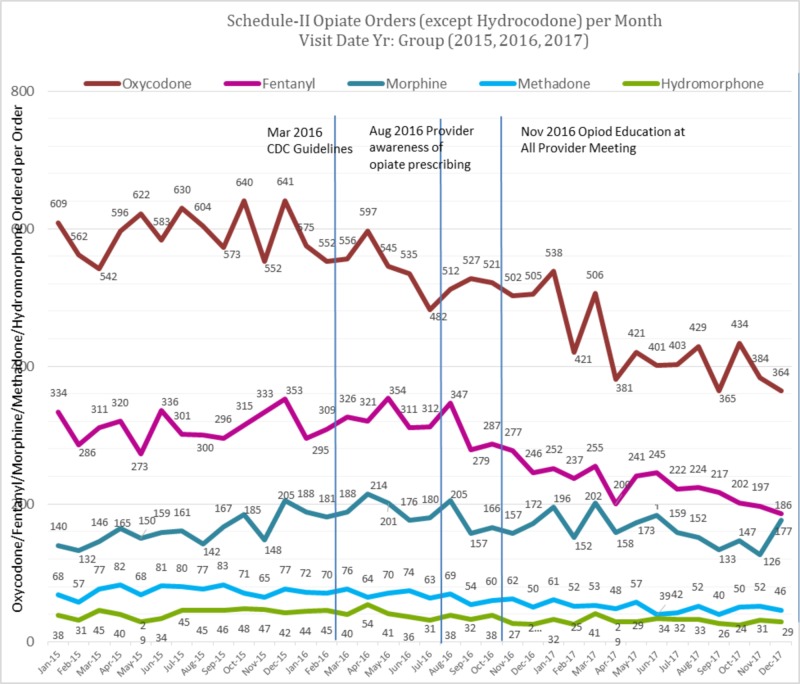
Schedule-II Opiate Orders for Oxycodone, Fentanyl, Morphine, Methadone and Hydromorphone per month from 2015-2017

The number of unique patients in 2015, 2016, and 2017 was 119,167, 120,516, and 121,174, respectively. Figures 1 & 2 show prescribing trends for total Schedule II opioids as well as specific opioids from January 2015 to December 2017.

 

The total Schedule II opioid prescription rate was 19.6% lower than the average of the baseline. Similarly, an 18.6% reduction of hydrocodone prescriptions, a 22.8% reduction of oxycodone prescriptions, a 25.9% reduction of fentanyl prescriptions, a 5.2% reduction of morphine prescriptions, a 29.2% reduction of methadone prescriptions, and a 27.5% reduction of hydromorphone prescriptions from average of the baselines was noted. Interrupted time series regression was used to examine the effect of an intervention on opioid prescriptions since the start of data collection by the Opioid Task Force (August 2016). Since the outcome variable is a count variable, a Poisson regression model was used. The Quasi-Poisson model was used to account for over-dispersion. The Durbin-Watson test was used to test for the first-order autocorrelation, which indicated the absence of serial autocorrelation, p =0.8443.

Four major variables were included in the model:

1. The outcome: total schedule-II opiate orders

2. The time: the time elapsed since the start of the year 2015.

3. The time is coded 0 before August 2016; time after August 2016 is coded as sequential number time periods.

4. A dummy variable indicating the pre-intervention period (coded 0) or the post-intervention (coded 1)

Table 1 shows that the level change right after the intervention is statistically significant. There is a reduction in total schedule-II opiate orders with a risk decrease of 7% [risk ratio (RR) 0.933; 95% confidence interval (CI) 0.874-0.996; p = 0.0373].

**Table 1:** Interrupted time series analysis

						95% CI	
	Estimate	StdErr	z	P_value	Risk ratio	Lower limit	Upper limit
Intervention	-0.06964	0.033	-2.08	0.0373	0.933	0.874	0.996
Time	-0.00004	0.002	-0.02	0.9806	1.000	0.996	1.004
Time after	-0.01777	0.003	-5.25	< .0001	0.982	0.976	0.989

**Figure 3:**
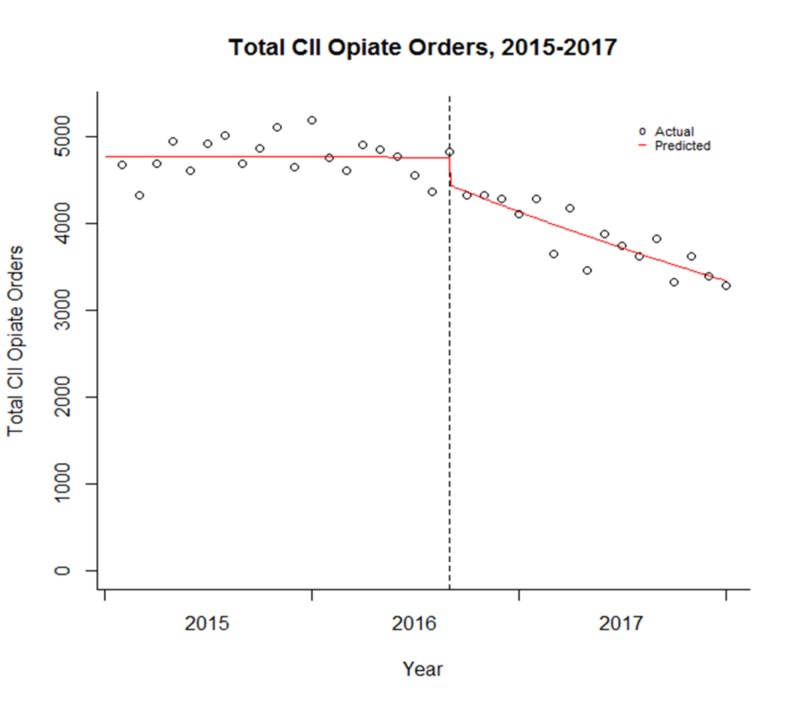
The total Schedule II opiate orders before and after the interventions for prescribing opioids The dashed vertical line represents August 2016.

As illustrated in Figure 3, the regression coefficient on “time after” captures the continuing effect of the interventions, that is, the slope of change in successive time periods.

The coefficient for time after is statistically significant, indicating that the downward trend in total schedule-II opiate orders is statistically significant. After August 2016, every month, there is a reduction of total schedule-II opiate orders with a risk decrease of 2% [RR 0.982; 95% CI 0.976-0.989; p < 0.0001].

**Figure 4:**
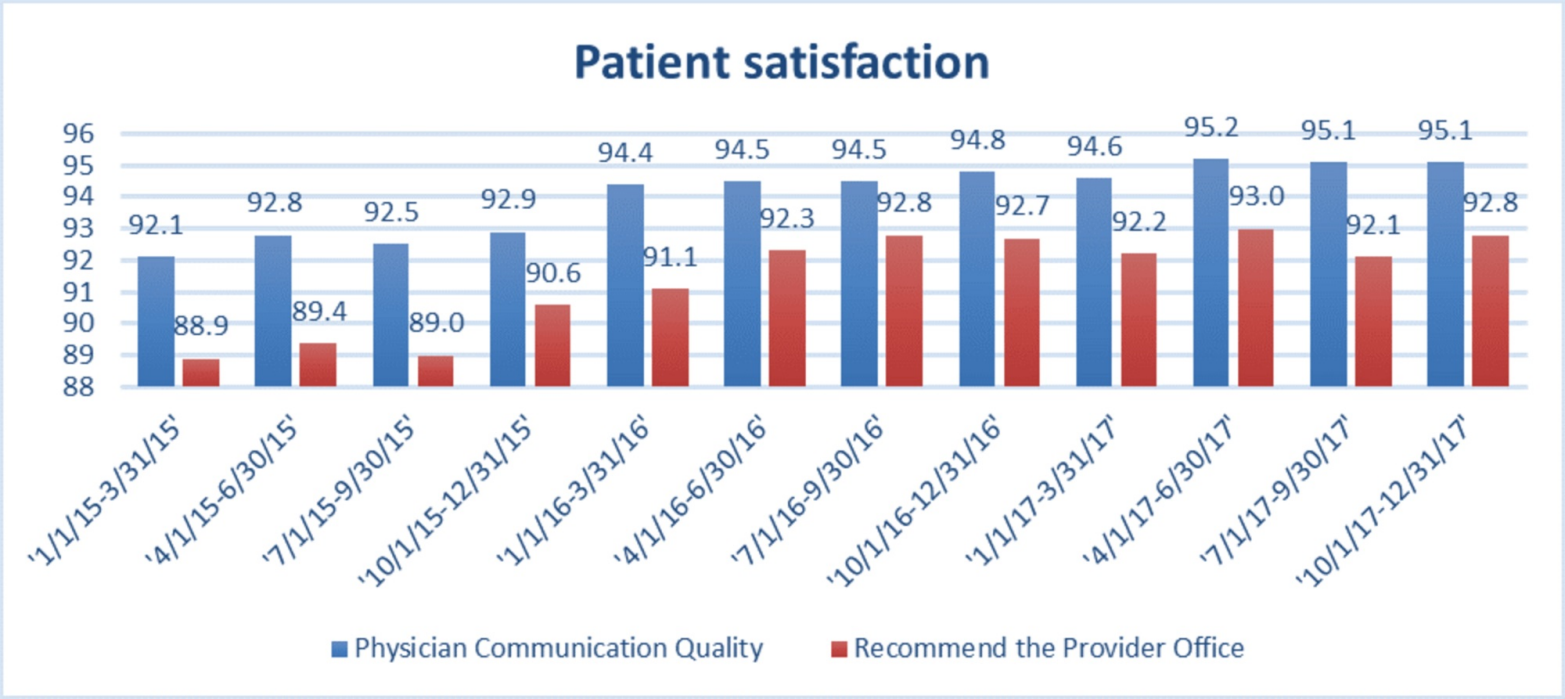
Patient satisfaction scores

**Figure 5:**
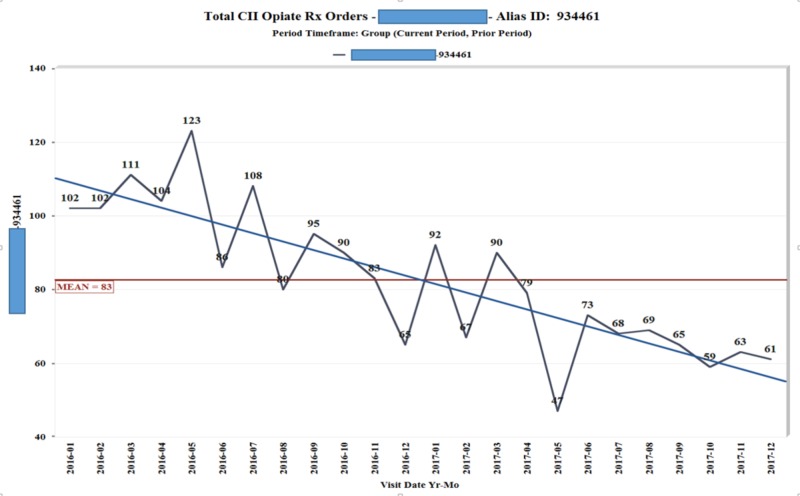
Sample provider report

**Figure 6:**
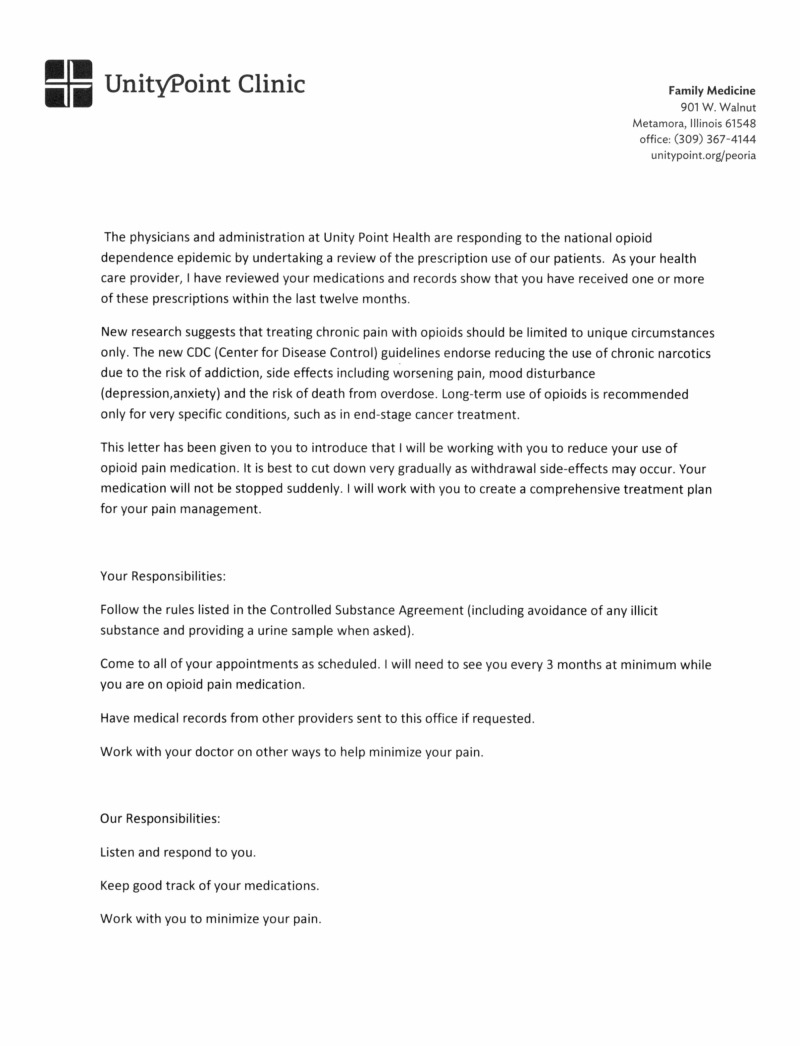
Patient education letter

Patient satisfaction scores improved during the reporting period (Figure 4).

## Discussion

Misuse, abuse, diversion, addiction, and overdose of opioids have created a serious public health epidemic in the U.S [22]. We noticed an initial decrease in opioid prescribing with the release of the CDC guidelines. However, a greater decline in opioid prescribing was noted after distributing data to providers that compared their own opioid prescribing patterns to their peers. There is variability among providers’ philosophies and practice patterns in accordance with the patients’ requirements and responses to the medications. This data comparing opioid prescribing patterns among providers alerted the clinicians because it could be seen that the amount of prescriptions written by some of them and the usual prescribing patterns of the majority of their colleagues differed. It offered an opportunity for self-analysis to clinicians to justify the clinical reasons for writing more opioid prescriptions: by reviewing the patients’ charts to evaluate the rationale of therapy, by determining its effectiveness in pain reduction and improvement in function, and by making referrals to specialists with more expertise, if appropriate. Similar monitoring of opioid prescribing practices had been described by a unique method named “In and out of the box [23].” In this article, Passik and Kirsh suggested that providers compare their own opioid prescribing patterns to their peers at regular intervals. The “In the box” method referred to the prescribing of opioids in a typical fashion like the prescribing patterns of their colleagues. In contrast, the “Out of the box” method referred to prescribing patterns that deviate from the usual prescribing habits of majority of providers treating chronic pain patients. The “Out of the box” prescribing served as an alert for the provider to be more rigorous in documentation and patient monitoring.

Our providers were educated to follow a multifaceted approach to monitor patients’ compliance with therapy to reduce opioid misuse and abuse. Compliance with opioid therapy has been found to be inadequate through behavior monitoring in chronic pain patients [24-25]. Hence, documentation of the controlled substance agreement for patients, urine drug testing (at baseline and at the minimum of one year), and reviewing prescription drug monitoring (before starting and during maintenance of opioid therapy) were recommended. Recent studies have shown the importance of provider education to decrease opioid prescribing [26-28]. We utilized the opportunity of presenting education in meetings that targeted a large audience (e.g. retreats, CME events, online modules) to fill the gap in knowledge for opioid prescribing and encourage collaboration with pain/addiction specialists to manage more complex cases.

Similar to a recent study conducted by Darnall, Ziadni, and Stieg, most of the gradual opioid tapering in our healthcare system was also done without behavior treatment unless the patient had substance abuse disorder [29]. Our providers were guided to collaborate with their patients by educating them about the benefits of opioid reduction and engaging them in shared decision making with a detailed plan of gradual opioid reduction with a close clinician follow-up. It is possible that some of the decreases in opioid prescribing observed here could be the result of the decrease in the appropriate use by the patients rather than improved patient care. However, the patient satisfaction scores improved during this transition period which suggested a successful patient-centered opioid tapering.

Our report has certain limitations. Opioid prescribing in the United States decreased overall after the release of the CDC 2016 Guidelines [30]. The interventions used to implement these guidelines were initiated as a bundle in our healthcare system. Hence, it is impossible to determine which intervention was most effective. There is no prospective controlled population. We could not assess the appropriateness of opioid therapy for individual patients. Indications for opioid therapy such as pain management for cancer pain or palliative care versus chronic non- cancer pain were not available. The major adverse events related to opioid use, such as opioid overdose deaths and opioid use disorder, were not measured in this data source. Even though we educated providers on a slow tapering process for opioid reduction, the incidence of opioid withdrawals related to the tapering was not measured.

## Conclusions

We describe a comprehensive approach to reduce opioid prescribing patterns and to influence the opioid epidemic. Educating clinicians about rational pain management and educating patients about the benefits of opioid reduction are very important aspects in achieving reductions in opioid prescriptions. Monitoring clinicians’ prescribing patterns in comparison to their peers and monitoring patients on chronic opioid therapy are the key to responsibly prescribing opioids. It is important for providers to collaborate with patients for a slow wean and with pain specialists for multimodal pain management and/or addiction specialists for patients with opioid abuse disorder. These methods can help healthcare systems in reducing opioid prescribing without negatively affecting patient satisfaction. Future healthcare initiatives can utilize similar methods to collect quality data via the electronic medical record to change prescribing patterns and evaluate interventions.
